# Psychosocial profile of children and adolescents followed in a pediatric musculoskeletal pain clinic

**DOI:** 10.1186/1546-0096-12-S1-P104

**Published:** 2014-09-17

**Authors:** Vânia Schinzel, Juliana Molina, Melissa Fraga, Vanessa Miotto e Silva, Maria Teresa Terreri, Claudio A Len

**Affiliations:** 1 Pediatrics, UNIFESP, São Paulo, Brazil

## Introduction

Amplified pain syndrome preferentially affects girls between the ages of 10-17 years. It is a disease with multiple causes that could be associated with major psychosocial disorders of patients and caregivers, affecting their quality of life. These psychosocial aspects can interfere intensifying the pain.

## Objectives

This is a transversal study with the objective of evaluating the quality of life of patients seen in a pediatric musculoskeletal pain clinic, drawing a panorama of the educational, social, and psychological aspects.

## Methods

25 patients from our Pediatric Musculoskeletal Pain Clinic were consecutively selected. The patients and their caregivers responded to the following questionnaires: Children’s Depression Inventory (CDI), PedsQL™ (Pediatric Quality of Life Inventory™) 4.0, PedsQL Multidimensional Fatigue Scale, family APGAR score, and SF-36 Health Survey.

## Results

We included 25 patients between the ages of 8 and 17, with an average of 12.6 years, 68% girls. In relation to the CDI, 95% were below the cutoff point, average=5.72. The highest score was 18 points. In the Family APGAR, the average was 13.4. In the PedsQL 4.0 the score of patients were between 20.8 and 95.1 with an average=62.6 and SD=19.8. The score from the point of views of patients were between 25.15 and 90.1, with an average-59.5 and SD=18.6. Regarding the PedsQL-Fatigue, the variation was between 20.8 and 91.7, with an average=60.9 and SD=17.4, and the caretakers varied between 18.0 and 93.0 with an average=60.6 and SD=22.3. In the SF-36 the patients obtained averages (SD) of 57.7(29.14); 43.2(47.14); 46.5(21.87); 47.4(20.88); 52.2(19.16); 54.4(28.46); 56.3(35.36), and 61.9(24.30) for the domains: Functional capacity, limitation from physical aspects, pain, general health, vitality, social aspects, emotional and mental health aspects, respectively.

## Conclusion

The patients did not present high rates of depression. However, we observed problems in family relationship and social life. Psychological problems could establish a causal relationship in some cases or reinforce the sensation of pain.

## Disclosure of interest

None declared.

